# Anterior Fusion Technique for Multilevel Cervical Spondylotic Myelopathy: A Retrospective Analysis of Surgical Outcome of Patients with Different Number of Levels Fused

**DOI:** 10.1371/journal.pone.0091329

**Published:** 2014-03-11

**Authors:** Shunzhi Yu, Fengning Li, Ning Yan, Chaoqun Yuan, Shisheng He, Tiesheng Hou

**Affiliations:** 1 Department of Orthopedics, Shanghai Tenth People's Hospital, Tongji University School of Medicine, Shanghai, China; 2 Department of Orthopedics, Third Affiliated Hospital of PLA Second Military Medical University, Shanghai, China; Toronto Western Hospital, Canada

## Abstract

**Objective:**

The anterior approach for multilevel CSM has been developed and obtained favorable outcomes. However, the operation difficulty, invasiveness and operative risks increase when multi-level involved. This study was to assess surgical parameters, complications, clinical and radiological outcomes in the treatment of 2-, 3- and 4-level CSM.

**Methods:**

A total of 248 patients with 2-, 3- or 4-level CSM who underwent anterior decompression and fusion procedures between October 2005 and June 2011 were divided into three groups, the 2-level group (106 patients), the 3-level group (98 patients) and the 4-level group (44 patients). The clinical and Radiographic outcomes including Japanese Orthopedic Association (JOA) score, Neck Disability Index (NDI) score, Odom's Scale, hospital stay, blood loss, operation time, fusion rate, cervical lordosis, cervical range of motion (ROM), and complications were compared.

**Results:**

At a minimum of 2-year follow-up, no statistical differences in JOA score, NDI score, Odom's Scale, hospital stay, fusion rate and cervical lordosis were found among the 3 groups. However, the mean postoperative NDI score of the 4-level group was significantly higher than that in the other two groups (P<0.05), and in terms of postoperative total ROM, the 3-level group was superior to the 4-level group and inferior to 2-level group (P<0.05). The decrease rate of ROM in the 3-level group was significantly higher than that in the 2-level group, and lower than that in the 4-level group (P<0.05).

**Conclusions:**

As the number of involved levels increased, surgical results become worse in terms of operative time, blood loss, NDI score, cervical ROM and complication rates postoperatively. An appropriate surgical procedure for multilevel CSM should be chosen according to comprehensive clinical evaluation before operation, thus reducing fusion and decompression levels if possible.

## Introduction

Cervical spondylotic myelopathy (CSM) is a common spinal cord disorder that develops in elderly individuals. Anterior cervical decompression and fusion is an effective and reliable procedure for the treatment of CSM [1]. However, multilevel CSM is a challenging clinical problem. The optimal surgical approach for multilevel CSM remains controversial. Anterior, posterior and combined anterior and posterior surgical approaches for patients with multilevel CSM all have been advocated [2]–[5]. Although laminectomy and laminoplasty have been effective for the treatment of multilevel cervical spondylotic myelopathy, progressive cervical kyphosis, C5 nerve root palsy, and axial neck pain are major disadvantages of these techniques [6]–[8]. The ventral aspect of the spinal cord and nerve roots is most often compressed [9]. Anterior cervical corpectomy and fusion better restores cervical lordosis and directly decompresses the spinal cord by removing the offending soft or hard discs [10]. This surgical procedure is widely used for the treatment of multilevel CSM [11]. Recent studies showed that multilevel anterior cervical discectomy and fusion (ACDF) and anterior cervical hybrid decompression and fusion (ACHDF) could obtain satisfying clinical efficacy in the management of multilevel CSM for appropriate patients [12]–[14]. In addition, discontinuous corpectomy and fusion (DCF) with reservation of the middle vertebra has been proved to be a safe and effective surgical treatment for multilevel CSM in our previous study [15].

The anterior approach treatment of 2-level CSM had been developed and obtained favorable outcomes [16], [17]. Some studies also exist regarding procedures involving 3 and 4-level segments [13]–[15], [18]. However, as number of fused and decompressed levels increased, the operation difficulty, invasiveness and operative risks are higher. Thus we decided to conduct this retrospective study to assess surgical parameters, complications, clinical and radiological outcomes in the treatment of 2-, 3- and 4-level CSM.

## Materials and Methods

### Patient Population

Two hundred and sixty patients who underwent surgery for 2-, 3- or 4-level CSM between October 2005 and June 2011 were included in the study. Twelve patients were excluded because they did not complete 2-year follow-up. The study group comprised 100 women and 148 men of median age 60.2 years (age range, 39–82 y). Radiological diagnoses were established in each patient via routine preoperative cervical anteroposterior, lateral, flexion-extension radiographs and cervical magnetic resonance imaging (MRI) or computed tomography (CT) scans. All patients had symptoms and signs of neural compression that were refractory to conservative treatment. The indication for number of surgical levels depended on several factors: extent of spinal cord compression, extent of the signal alteration of the spinal cord in the MRI, segmental and cervical alignment. The exclusion criteria were as follows: we excluded patients whose primary symptoms were axial pain or radicular symptoms, and not myelopathy symptoms. Severe stenosis patients were excluded (Pavlov ratio less than 0.70). Patients with severe ischemic heart disease, Lung disorders and blood dyscrasias were excluded. Patients who underwent prior cervical spine surgery or underwent surgery for fractures, tumors, or infection were also excluded. According to the segments involvement, these patients were divided into three groups, the 2-level group (106 patients), the 3-level group (98 patients) and the 4-level group (44 patients).

### Surgical Technique

All operations were performed by an experienced spine surgeon (H.T.) with more than 30 years of clinical experience in cervical spine surgery. The choice of the operation was dependent on the characteristics of cord compression. Large osteophyte and disc complexes extending posterior to the vertebral body were decompressed by corpectomy. If the compression is caused by the anterior degenerative discs, discectomy is performed. Anterior cervical corpectomy and discectomy were performed as described previously [13], [19], [20]. The operative procedure for DCF has been described by our previous study [15]. The ACHDF procedure included 1-level ACDF and 1-level ACCF. For complete decompression, the posterior longitudinal ligament was also removed to expose the dura throughout the length of the corpectomy and discectomy. For the corpectomy procedures, cartilaginous end plates were removed from the adjoining vertebral bodies. The corpectomized bone was harvested, morsellized, and packed into appropriately sized titanium mesh (DePuy Spine, New Brunswick, New Jersey). For discectomy procedure, PEEK interbody cage (DePuy Spine, New Brunswick, New Jersey) was used to fill the space generated by discectomy. Finally, an appropriately sized anterior cervical locking plate was firmly fixed into the vertebrae with screws (DePuy Spine, New Brunswick, New Jersey).

**Figure 1:**
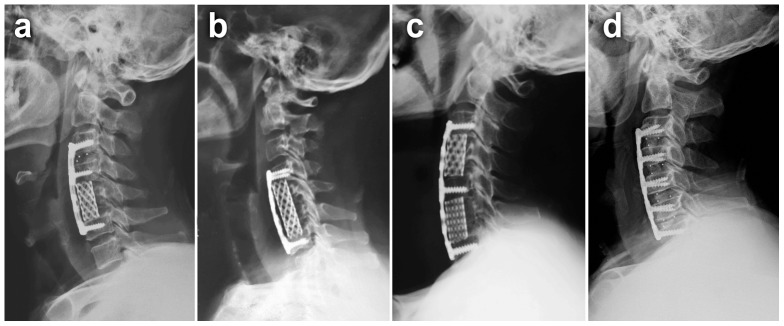
Postoperative lateral radiographs of four surgical techniques. **a** Anterior cervical hybrid decompression and fusion (ACHDF). **b** Anterior cervical corpectomy and fusion (ACCF). **c** Discontinuous corpectomy and fusion (DCF) with reservation of the middle vertebra. **d** Anterior cervical discectomy and fusion (ACDF).

When CSM was 2-level, 2-level ACDF, or 1-level ACCF was performed. When CSM was 3-level, 3-level ACDF, ACHDF or 2-level ACCF was performed. When CSM was 4-level, 4-level ACDF or DCF was performed. In the 2-level group, 42 patients underwent ACCF, 62 patients underwent ACDF. In the 3-level group, 18 patients underwent ACCF, 33 patients underwent ACDF, and 47 patients underwent ACHDF. In the 4-level group, 19 patients underwent ACDF, 25 patients underwent DCF(Table 1). All patients wore Philadelphia collars for an average of 10 weeks postoperatively (Fig. 1).

**Table 1:** Showing number of different procedures in three groups.

	2level	3level	4level
ACCF	42	18	0
ACDF	64	33	19
DCF	0	0	25
ACHDF	0	47	0
total	106	98	44

ACCF: anterior cervical corpectomy and fusion;

ACDF: anterior cervical discectomy and fusion;

DCF: Discontinuous corpectomy and fusion (DCF) with reservation of the middle vertebra;

ACHDF: anterior cervical hybrid decompression and fusion.

### Ethics Statement

The clinical investigation has been conducted according to the principles expressed in the Declaration of Helsinki. All of the patients signed the informed consent form before their information was stored in the hospital database and used for research purposes. The study was approved by the Ethical Committee of the Tenth People's Hospital of Tongji University.

### Outcome Measures

**Figure 2:**
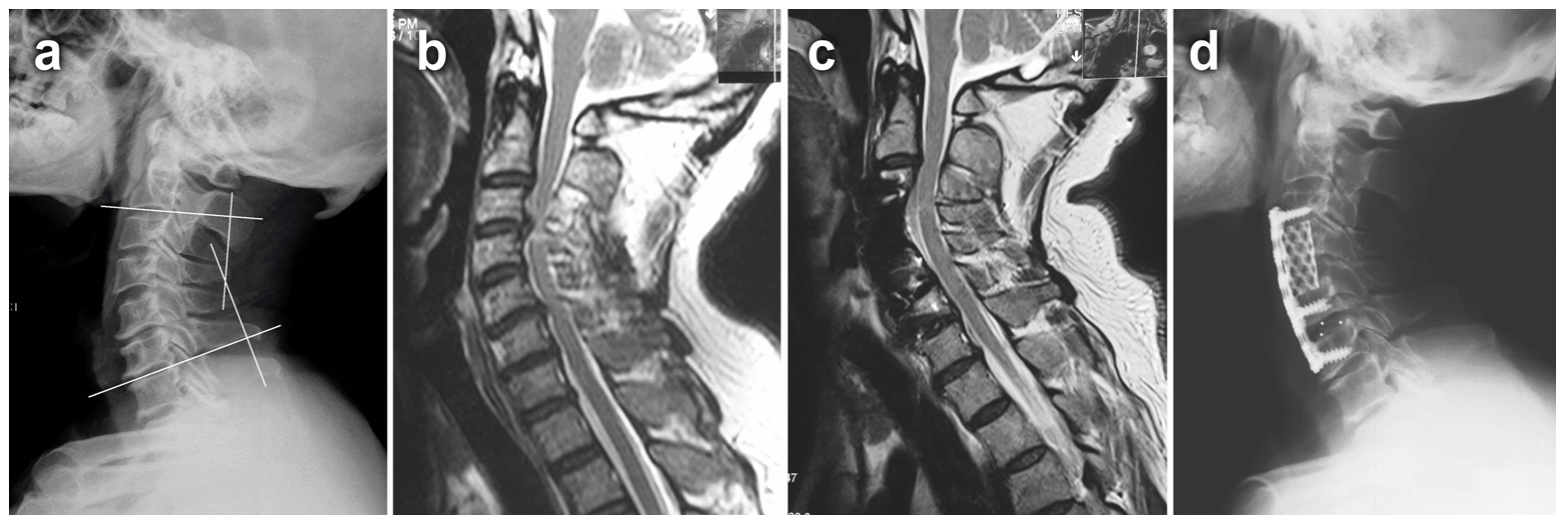
A 65-year-old male developed numbness in his two hands and weakness in his four extremities for 2 years. Preoperative imaging studies showed that the spinal cord compressed at C3–C6. He was performed with 3-level ACHDF. After operation, his JOA scores improved from 7 preoperation to 13 postoperation. **a** Preoperative lateral X-ray. The segmental lordosis of C2–C7 was defined as the angle formed by the lower endplate of C2 vertebral body and the upper endplate of C7 vertebral body. **b** Preoperative MRI. **c** 2-year postoperative MRI. **d** 2-year postoperative lateral X-ray.

Neurological function was assessed using the Japanese Orthopedic Association (JOA) scores. Postoperative patients' satisfaction was based on Odom's criteria. The Neck Disability Index (NDI) score was also recorded for the evaluation of neck-shoulder pain levels. The degrees of preoperative and final follow-up fusion segmental lordosis of C2–C7 was measured using the Cobb method described in previous study [5] (Fig. 2). Lateral radiographs in flexion and extension were assessed before and after surgery. Total cervical range of motion (ROM) was defined as the angle formed between the lower endplate of the C2 and the upper endplate of the C7 using Cobb's method on flexion/extension lateral radiographs [21]. The decrease rate of ROM after the operation was calculated by [(pepreoperative ROM – postoperative ROM)/preoperative ROM] ×100%. Bone fusion was judged by the absence of motion more than 2° between the spinous processes on flexion–extension lateral radiographs, the absence of radiolucent gap between the graft and end plate, and the presence of continuous bridging bony trabeculae at the graft-endplate interface. Movement of ≥2° on flexion/extension radiographs was regarded as a pseudarthrosis [22], [23]. Graft dislodgement was defined as graft beyond the leading edge of the upper and lower vertebral connection 2–4 mm on lateral radiographs. The graft subsidence was defined as loss of height of the fusion segments on lateral radiographs at day 1 after the surgery and at bony fusion. The incidence of dysphagia was defined as that solid or dry food gets stuck in the process of swallowing.

The following data were recorded for each patient: history; symptoms at admission; duration of symptoms; physical and neurological findings at presentation; intraoperative spinal observations; preoperative and postoperative neurological function; preoperative and postoperative radiological findings and intra- and postoperative complications. Postoperative follow-up visits were done regularly at 3months, 6 months, 12 months 18 months and 2 years. The subsequent follow-up examinations were performed at every 6-month interval. All the patients were observed for at least 24 months after surgery.

### Statistical Analysis

Data were analyzed using Microsoft Excel 2003 (Microsoft, Redmond, Washington) and SPSS version 19.0 software (SPSS, Inc, Chicago, Illinois). The changes in clinical effects and cervical lordosis in each group after surgeries were analyzed by the Wilcoxon rank-sum test. The Kruskal-Wallis *H* test was used to investigate whether the statistical differences of results exist among the groups postoperatively and post-hoc analysis was performed using the Nemenyi test. The chi-square test was used in the comparisons of the complication among the groups. A P value less than 0.05 was considered statistically significant.

## Results

### Perioperative Parameters

The overall follow-up period of the patients ranged from 24 to 48 months (average 29.2 months). The duration of symptoms at presentation ranged from 6 months to 4 years (median, 1.6 years). Major symptoms at presentation were arm numbness or paresthesias (n = 194; 78.2%), neck and arm pain (n = 172; 69.4%), motor weakness (n = 155; 62.5%), and gait disorder (n = 89; 35.9%). Most patients showed various degrees of symptom relief postoperatively. The general information was presented in Table 2. No significant intergroup differences were found in terms of age, gender and hospital stay (P>0.05). The mean operative time and blood loss in the 2-level group were significant lower than the 3-level group (P<0.05). The 4-level group required a significantly longest operative time than the other two groups (P<0.05) and had more operative blood loss (P<0.05).

**Table 2:** Demographic data of patients.

	2-level group (n = 106)	3-level group (n = 98)	4-level group (n = 44)	P
**Age (year)**	59.24±9.60	60.60±9.84	61.50±10.00	0.451
**Gender (Male/Female)**	63/43	60/38	25/19	0.883
**Active smokers(yes/no)**	33/73	36/62	14/30	0.676
**Patient with diabetes**	19	21	5	0.354
**Patient with hypertension**	22	23	11	0.821
**Hospital stay (day)**	11.71±2.67	11.59±3.00	12.41±2.56	0.074
**Operative time (min)**	106.51±17.90*	129.64±16.87*	166.14±20.65*	**0.000**
**Blood loss (mL)**	126.42+28.86#	154.00±30.32#	194.77±42.34#	**0.000**

Statistical significance was set at a P<0.05.

The Kruskal-Wallis *H* test was used to investigate whether the statistical differences exist among the groups.

*Operative time: P = 0.000 (2-level and 3-level groups); P = 0.000(2-level and 4-level groups); P = 0.000 (3-level and 4-level groups) by Nemenyi test.

# Blood loss: P = 0.000 (2-level and 3-level groups); P = 0.000(2-level and 4-level groups); P = 0.000 (3-level and 4-level groups) by Nemenyi test.

### Clinical and Radiological Outcomes

Table 3 showed the Clinical and Radiological Results. The Japanese Orthopaedic Association scores significantly increased from 8.80±1.05 to 12.84±1.34 in the 2-level group (P<0.05), from 8.52±1.18 to 12.83±1.59 in the 3-level group (*P*<0.05), and from 8.45±1.15 to 12.41±1.47 in the 4-level group (P<0.05), respectively. There were no significant differences in JOA scores and Odom criteria among the 3 groups (P>0.05). In terms of NDI score, total cervical ROM and cervical lordosis, no significant intergroup difference was found preoperatively (P>0.05). NDI score, total cervical ROM and cervical lordosis were statistically different between preoperation and postoperation in each group (P<0.05). There were no significant differences in cervical lordosis among the 3 groups. However, in terms of NDI score, the mean postoperative NDI score of the 4-level group was significantly higher than that in the other two groups (P<0.05), and in terms of postoperative total ROM, the 3-level group was superior to the 4-level group and inferior to 2-level group (P<0.05). The decrease rate of ROM in the 3-level group was significantly higher than that in the 2-level group, and lower than that in the 4-level group (P<0.05). Postoperative radiographs demonstrated that fusion rates were 89.6% in the 2-level group, 91.2% in the 3-level group and 87.8% in the 4-level group at 3 months postoperatively. The fusion rates were 96.2% in the 2-level group, 96.9% in the 3-level group and 97.2% in the 4-level group at 24 months postoperatively.

**Table 3:** Comparisons of Clinical and Radiographic Outcomes.

	2-level group (n = 106)	3-level group (n = 98)	4-level group (n = 44)	P
Preoperative JOA scores	8.80±1.05	8.52±1.18	8.45±1.15	0.108
JOA scores at the final follow-up	12.84±1.34	12.83±1.59	12.41±1.47	0.286
Preoperative NDI scores	21.77±3.97	21.58±4.20	22.20±3.51	0.708
NDI scores at the final follow-up	11.63±1.62	12.12±2.22	13.27±2.17*	**0.000**
Odom's Scale (Excellent/good/fair/bad)	14/39/42/11	18/36/39/5	4/16/20/4	0.443
Preoperative Segmental lordosis (degree)	12.40±1.96	12.16±3.23	11.61±4.21	0.780
Segmental lordosis at the final follow-up (degree)	20.44±2.27	20.32±2.46	19.84±2.88	0.685
Preoperative ROM (degree)	43.31±3.61	43.08±4.11	42.55±3.43	0.646
ROM at the final follow-up (degree)	36.06±3.37#	34.65±4.20#	29.80±2.33#	**0.000**
Decrease rate of ROM (%)	16.72±3.71▴	19.67±4.51▴	29.71±6.04▴	**0.000**

JOA Japanese orthopedic association; NDI neck disability index; ROM, range of motion.

Statistical significance was set at a P<0.05.

The Kruskal-Wallis *H* test was used to investigate whether the statistical differences exist among the groups.

*NDI scores at the final follow-up: P = 0.078 (2-level and 3-level groups); P = 0.000(2-level and 4-level groups); P = 0.002 (3-level and 4-level groups) by Nemenyi test.

# ROM at the final follow-up: P = 0.005 (2-level and 3-level groups); P = 0.000(2-level and 4-level groups); P = 0.000 (3-level and 4-level groups) by Nemenyi test.

▴ Decrease rate of ROM: P = 0.005 (2-level and 3-level groups); P = 0.000(2-level and 4-level groups); P = 0.000 (3-level and 4-level groups) by Nemenyi test.

### Complications

In the 2-level group, a total of 12 (11.3%) patients developed postoperative complications including dysphagia (3 cases), dysphonia (2 cases), C5 palsy (0 cases), cerebral fluid leakage (2 case), pseudarthrosis (2 case), graft displacement (1 case), and subsidence (2 cases). In the 3-level group, a total of 18 (18.4%) patients developed postoperative complications including dysphagia (6 cases), dysphonia (3 cases), C5 palsy (1 cases), cerebral fluid leakage (2 case), pseudarthrosis (2 case), graft displacement (1 case), and subsidence (3 cases). In the 4-level group, a total of 17 (38.6%) patients developed postoperative complications including dysphagia((8 cases), dysphonia (2 cases), C5 palsy (1 case), cerebral fluid leakage (2 case), pseudarthrosis (1case), and subsidence (3 cases). The patient with pseudarthrosis was asymptomatic and did not receive second surgery. Cerebrospinal fluid leakage usually stopped after 3 to 5 days of conservative treatment with local pressure. The graft displacement and subsidence complications all occurred in patients with titanium mesh (ACCF, ACHDF and DCF). Statistical analysis showed that the 3-level group had higher incidence of postoperative complications than the 2-level groups, and the postoperative complications incidence of the 4-level group is highest (P<0.05) (Table 4).

**Table 4:** Complications.

	2-level group (n = 106)	3-level group (n = 98)	4-level group (n = 44)
Dysphagia	3(2.8%)	6(6.1%)	8(18.2%)
Dysphonia	2(1.9%)	3(3.1%)	2(4.5%)
C5 palsy	0	1(1.0%)	1(2.3%)
CSF leakage	2(1.9%)	3(3.1%)	2(4.5%)
Pseudarthrosis	2(1.9%)	1(1.0%)	1(2.3%)
Graft displacement	1(1.0%)	1(1.0%)	0
Subsidence	2(1.9%)	3(3.1%)	3(6.8%)
Total	12(11.3%)*	18(18.4%)*	17(38.6%)*

Statistical significance was set at a P<0.05.

The chi-square test was used in the comparisons of the complication among the groups.

*Total: P = 0.038 (2-level and 3-level groups); P = 0.010(2-level and 4-level groups); P = 0.000 (3-level and 4-level groups).

## Discussion

Anterior cervical decompression and fusion was first reported by Robinson and Smith [24] and popularized by Cloward [25] in the 1950s. The advantages of anterior decompression are the direct decompression and resection of the object causing pressure on the spinal cord in front, including soft disc herniations backwards, osteophytic proliferation, and ossification of the posterior longitudinal ligament. Although the surgical treatment of multilevel CSM is associated with less predictable outcomes and a higher frequency of complications, anterior approach in multilevel procedures is beneficial [13].

According to our retrospective review of 248 patients, all the three groups demonstrated a significant increase in JOA scores that were maintained at the finally follow-up. The clinical outcomes showed no significant differences among the three groups. The differences of Odom criteria and cervical lordosis were not statistically significant either. However, significant differences were observed in NDI score and total cervical ROM. The NDI score was significantly higher in the 4-level group than that in the other two groups. Wu et al. [26] found that patients might not experience great difficulties in performing daily activities. However, in our study, as number of fused levels increased, a small part of patients still complained of different degrees of neck pain and stiffness during follow up. We believed that this difference may be due to vertebral excessive distraction during operation [27]. Disc degeneration is usually more serious in patients with multi-level CSM. Intervertebral disc space is too narrow because of subsidence. Surgeons might habitually turn the lever of the Caspar retractor without considering how much excessive force is being applied to the vertebra for the purpose of physiological lordosis restoration and intervertebral disc space exposure. Some studies have proved that the mechanical load on the facet joints caused neck pain [28], [29]. Overdistraction could cause posterior neck pain due to stretching of the facet joint [29], [30]. Therefore, we hypothesized that vertebral excessive distraction might be related to subsequent neck pain. In addition, the total cervical ROM was significantly decreased when more levels had been fused. we found that the loss of neck motion after fusion also made patients feel depressed and anxious subjectively during follow up, especially the patients with 4-level fusion.

No surgeon can afford to neglect the complications of cervical procedures. As involved segments increased, the incidence of operative complications increased, too [31]–[36]. Kang et al. [37] reported the risk of dysphagia was greater in the group who underwent multilevel rather than single level surgery. Danto et al. [32] also showed that the risk of developing dysphagia and/or dysphonia increases with the number of surgical levels. In our study, we also found the 3-level and 4-level group had higher incidence of postoperative complications than the 2-level groups. A right-sided, 5 cm transverse straight incision was used in our surgical procedure so as to reduce the exposure area and retractor pressure on the esophagus. Tracheal/esophageal traction exercise (TTE) treatment [38] was also used preoperatively. However, there were still 14 patients feeling dysphagia and 5 patients feeling dysphonia in various degrees after surgery in the 3-level and 4-level group, and only 3 patients feeling dysphagia and 2 patients feeling dysphonia after surgery in the 2-level group. This kind of complication seems cannot be easily prevented when the number of fused levels increased. Meanwhile, there was a trend toward higher rate of dysphagia and dysphonia in 4-level group than 3-level group.

**Figure 3:**
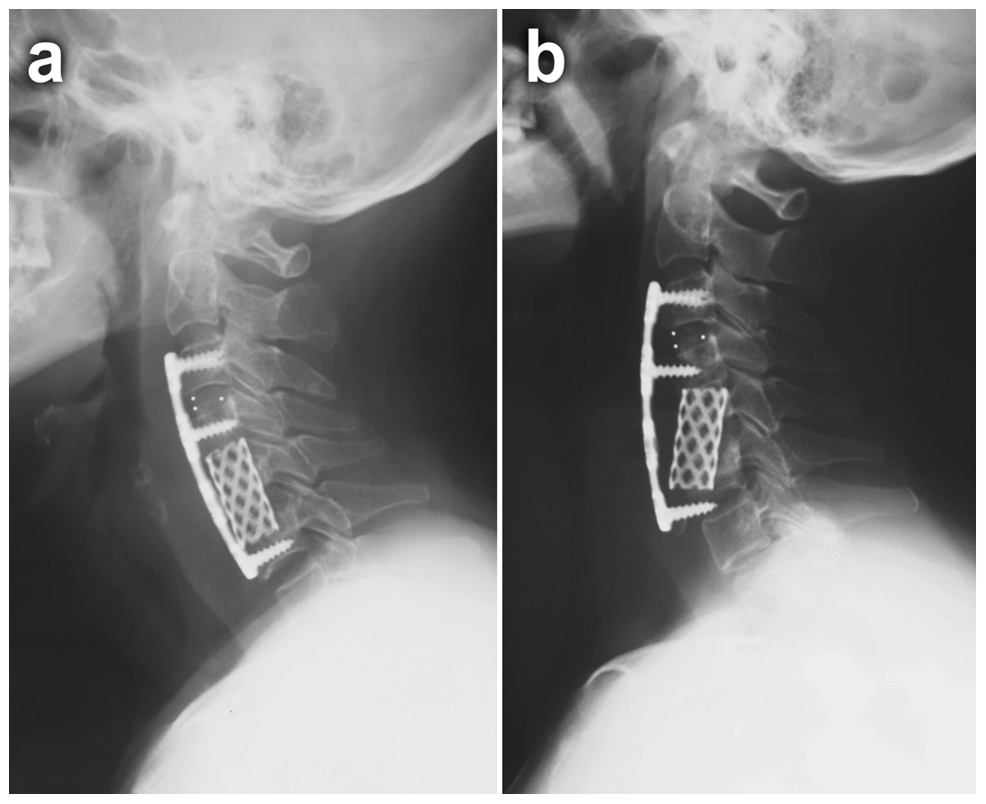
A 62-year-old female patient with cervical spondylotic myelopathy at the C3–C6 level. She was performed with 3-level ACHDF. The postoperative lateral view of this patient showed displacement of the titanium mesh cage and screw loosening at the three months follow-up. **a** Immediate postoperative lateral X-ray. **b** 3-month postoperative lateral X-ray.

Bone- and/or plate-related complications after multilevel corpectomy have been reported in high rates, even when internal fixation was used [39]. Failure rates increase with 3 or more levels of corpectomy [33]. In the present study, there were 1 case of graft displacement and 2 cases of subsidence in the 2-level group, 1 case of graft displacement and 3 cases of subsidence in the 3-level group, and 3 cases of subsidence in the 4-level group. All these patients had undergone corpectomy (ACCF, ACHDF and DCF), and none of patients with ACDF developed this kind of complications (Fig. 3). Liu et al. [5] reported that multilevel discectomy and fusion offer more fixation points to hold the construct rigidly in place. The failure rate is lower than corpectomy, especially in terms of graft dislodgement and subsidence. The choice of the operation procedure was dependent on the characteristics of cord compression in our study. Because large osteophyte and disc complexes extending posterior to the vertebral body may not be easily removed by discectomy, corpectomy is more suitable in such cases. Over all, if the compressive pathology could be resolved by discectomy, ACDF should be the treatment of choice. If subsidence of the intervertebral space is serious and the compression is mainly caused by the osteophyte or nucleus pulposus bulges out beyond the damaged posterior longitudinal ligament and extending posterior to the vertebral body. We usually select the ACHDF or DCF procedures. A long corpectomy was the last choice considered.

Furthermore, the controversy of multi-level CSM is not only the surgical approach, but also the selection of fused and decompressed levels. The choice of surgical procedure for treatment should be dictated by several factors, including the location of compression, extent of the pathology, the patient's chief complaint and medical condition. The complete cervical spine radiography, CT and MRI were preformed in all patients before surgeries. However, it is difficult to confirm if all the herniated discs are related to the symptoms of the patients at times. The operated segments might be chosen unilaterally according to the imaging examination. In our study, we also observed that operation time and blood loss both increased when more segements were fused, and the operation difficulty, invasiveness and operative risks were higher. Kou et al. [40] reported that multilevel surgical procedure was established a significant risk factor for epidural hematoma after operation. Grabowski et al reported that complex anterior cervical surgery had higher risk of esophageal and vertebral artery injuries [41]. Sagi et al. [42] also found that prolonged procedures exposing more than three vertebral levels that include C2, C3, or C4 with more than 300-mL blood loss should be watched carefully for respiratory insufficiency. In our study, we mainly focused on surgical results of patients with different number of operated levels. An appropriate surgical procedure for multi-level CSM should be chosen according to comprehensive clinical evaluation. Good decompression is necessary for optimal surgical outcome. Furthermore, reduce fusion and decompression segments so as to minimize operation trauma and surgical risks.

This study had some limitations. First, the investigation was retrospective study. The patient's number in 4-level group was relative small. Second, different surgical procedures performed in the same group might have influence on the fusion rate and Instrumentation and graft related-complications. Finally, the incidence of adjacent segment disease cannot be followed adequately because the follow-up period was a minimum of 2 year. Therefore, a longer randomized controlled trial study is needed.

## Conclusion

On the basis of our findings, we can conclude that surgical results become worse in terms of operative time, blood loss, postoperative NDI score, postoperative cervical ROM and complication rates when the number of involved levels increased. An appropriate surgical procedure for multi-level CSM should be chosen according to comprehensive clinical evaluation before operation, thus reducing fusion and decompression levels if possible.
